# The Use of Liquid Biopsy in the Molecular Analysis of Plasma Compared to the Tumour Tissue from a Patient with Brain Metastasis: A Case Report

**DOI:** 10.3390/medicina59030459

**Published:** 2023-02-25

**Authors:** Veronica Aran, Vinicius Mansur Zogbi, Renan Lyra Miranda, Felipe Andreiuolo, Nathalie Henriques Silva Canedo, Carolina Victor Nazaré, Paulo Niemeyer Filho, Vivaldo Moura Neto

**Affiliations:** 1 Laboratório de Biomedicina do Cérebro, Instituto Estadual do Cérebro Paulo Niemeyer, Rua do Rezende156-Centro, Rio de Janeiro 20231-092, Brazil; 2 Neurosurgery Division, Instituto Estadual do Cérebro Paulo Niemeyer, Rua do Rezende156-Centro, Rio de Janeiro 20231-092, Brazil; 3 Neuropathology and Molecular Genetics Laboratory, Instituto Estadual Do Cérebro Paulo Niemeyer, Rua do Rezende156-Centro, Rio de Janeiro 20231-092, Brazil

**Keywords:** brain metastases, lung hepatoid adenocarcinoma, *K-RAS*, liquid biopsy, ddPCR

## Abstract

Different cancers have multiple genetic mutations, which vary depending on the affected tumour tissue. Small biopsies may not always represent all the genetic landscape of the tumour. To improve the chances of identifying mutations at different disease stages (early, during the disease course, and refractory stage), liquid biopsies offer an advantage to traditional tissue biopsy. In addition, it is possible to detect mutations related to metastatic events depending on the cancer types analysed as will be discussed in this case report, which describes a patient with brain metastasis and lung cancer that harboured *K-RAS* mutations both in the brain tumour and in the ctDNA present in the bloodstream.

## 1. Introduction

The minimally invasive technique known as liquid biopsy can detect tumour-derived biomarkers in body fluids such as in blood. Circulating tumour DNA (ctDNA) refers to DNA of tumour origin, and being cell-free [1]. In fact, there are two sources of DNA that can be noninvasively assessed in blood circulation: cell-free circulating DNA (cfDNA) and the tumour-derived DNA fraction (ctDNA). They consist of small fragments of nucleic acid that are not associated with cells or cell fragments, and several studies have shown that ctDNA is present in advanced neoplasia [2]. This probably occurs because of apoptosis, cell necrosis, and active tumour secretion since tumour cell division occurs faster than normal cell division [3]. Thus, analysis of ctDNA is considered helpful in the prognosis, identification of alterations in different genes, selection of targeted therapies, and disease monitoring in cancer [4]. In addition, apart from blood (serum or plasma), sources of cfDNA/ctDNA include urine, saliva, cerebrospinal fluid, seminal fluid, and pleural fluid samples [3,5].

Plasma offers the possibility to give crucial molecular information via the analysis of ctDNA to detect cancer-related mutations in distinct tumour types, such as already described in the profiling of lung, colorectal, brain, and breast tumours, showing a promising perspective for clinical monitoring [6,7,8,9]. The first Food and Drug Administration (FDA) approval of a ctDNA liquid biopsy test occurred in 2016, being developed to detect epidermal growth factor receptor (*EGFR*) gene mutations in patients with non-small-cell lung cancer (NSCLC) as a companion diagnostic test. In recent years, multigene panel assays of liquid biopsy have been approved as companion diagnostics taking advantage of NGS methods for sequencing analyses [10]. The European Society for Medical Oncology (ESMO) guidelines recommend validated liquid biopsies for genotyping of patients with advanced cancer even when tissue-based testing remains the gold standard procedure, due to limitations of ctDNA assays in detecting certain molecular alterations [11]. Nevertheless, ctDNA analysis is an important choice when tissue biopsies are not possible [11].

The anatomical site of tumours was shown to correlate with variable amounts of ctDNA levels in plasma, with brain tumours showing the lowest levels probably due to the presence of the blood−brain barrier (BBB) [2,12]. The brain is composed of unique cells that perform tissue-specific functions (e.g., neurons, astrocytes, oligodendrocytes, etc). Brain tumours are heterogeneous, and depending on the tumour type, are considered among the most lethal, such as glioblastoma [13]. Brain metastases are one of the most frequent intracranial lesions, which develop via tumour cells passing from the bloodstream to the central nervous system through the breakdown of the BBB, resulting in distribution throughout the central nervous system, especially in areas of blood flow [14].

The colonisation of tumour cells from the primary tumour site into distant organs results in the formation of metastatic tumours. With regards to frequent metastatic sites in the body, usually the most common are the lung, liver, and brain, which were shown to present organ-specific patterns, brain metastases being one of the most lethal among them [15]. When comparing the genetic landscape of primary versus metastatic tumours, some studies have reported genetic heterogeneity in paired primary tumours and brain metastases [16], while others revealed high similarity among them [17]. In that sense, through the search for ctDNA mutations in different brain cancer patients that undergo surgery in our Institute, we detected *K-RAS* mutations in one patient’s plasma sample. This was unusual since *K-RAS* mutations are not frequently found in primary brain tumours but are, for example, commonly present in lung tumours, being also considered a therapeutic target in lung cancer. A recent study showed that in 242 gliomas analyzed for RAS gene alterations, only 3 showed *K-RAS* mutations confirming its infrequency [18]. Therefore, we gathered both molecular and clinical information to report this case.

The patient arrived at our Institute, transferred from an emergency care unit with an image of an expansive lesion on her occipital and parietal lobe and an infiltrative lesion on the occipital bone. After careful evaluation of the patient’s clinical and molecular data, the case described in this report was considered to represent an interesting example of the importance of liquid biopsy being performed alongside conventional biopsy. This case study was approved by the Human Ethics Committee of the *Instituto Estadual do Cérebro Paulo Niemeyer* (protocol N^o^ CAAE 90680218.6.0000.8110). The patient’s case will be discussed in this article as follows.

## 2. Case Report

**Figure 1:**
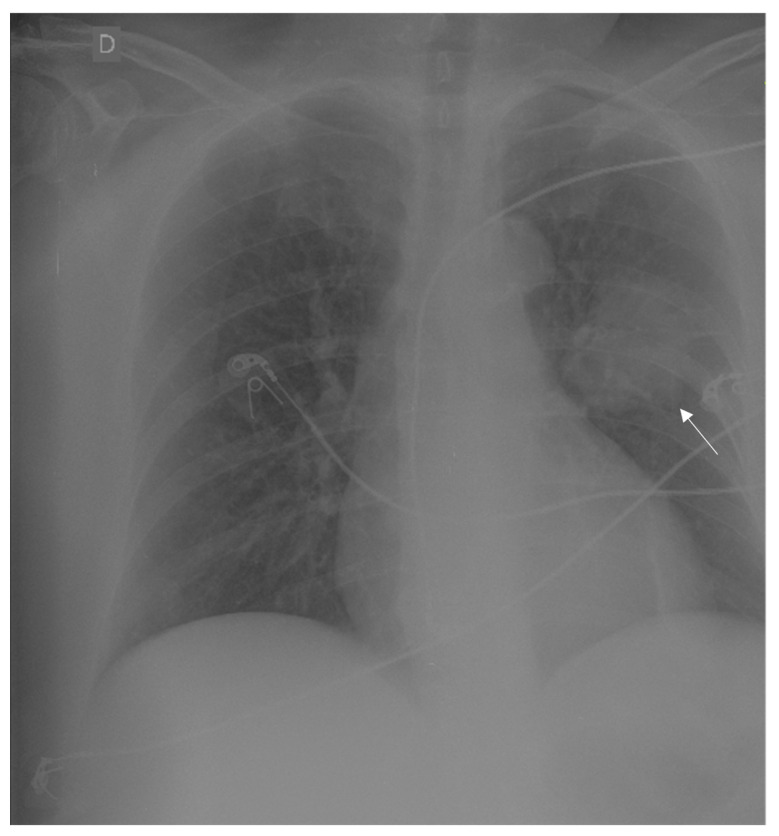
Chest X-ray showing a round mass in the upper lobe of the left lung (indicated with an arrow) suggestive of lung cancer (D in the image means right side).

A female, sixty-year-old patient presented with syncope, a history of headache, paresis in the right side of the body, and an occipital bulge with progressive growth in the past nine months. After being admitted to the first hospital, she underwent a chest x-ray that showed a pulmonary mass on the left side (Figure 1). Regarding clinical history, the patient was an ex-smoker with a smoking load of 1 pack/year for 40 years, with systemic arterial hypertension, dyslipidaemia, and a history of an acute heart infarct treated with stent placement 2 years before. No further investigation was performed at that time. She was then transferred to the *Instituto Estadual do Cérebro Paulo Niemeyer* (Rio de Janeiro, Brazil) for further evaluation and surgical programming.

**Figure 2:**
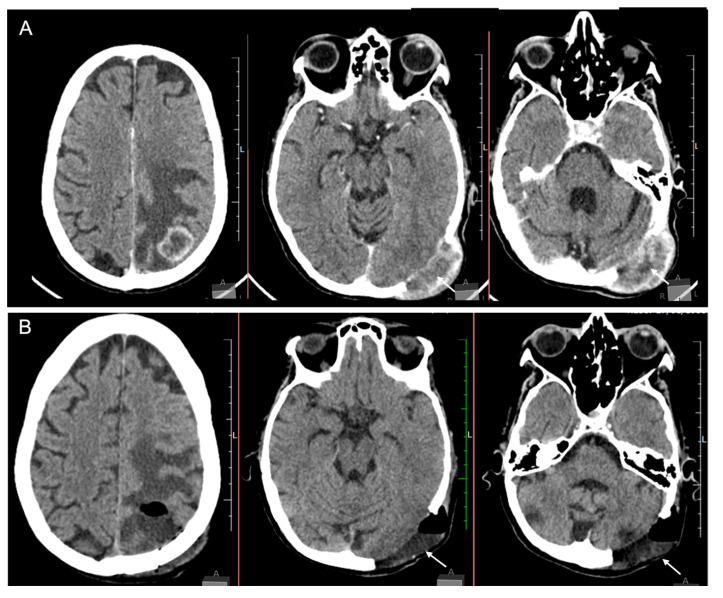
Computer tomography (CT scan) of the brain showing pre- (**A**) and postoperative images (**B**). (**A**) CT scan with venous contrast showing an intra-axial lesion on the occipital/parietal lobes and an infiltrative transcranial lesion in the occipital bone. (**B**) Postoperative image of both intracranial and transcranial lesions.

A brain CT scan revealed a parietal-temporal-occipital (PTO) lesion and a transcranial tumour (Figure 2). She underwent the surgical procedure to remove both lesions resulting in deficit improvement.

**Figure 3:**
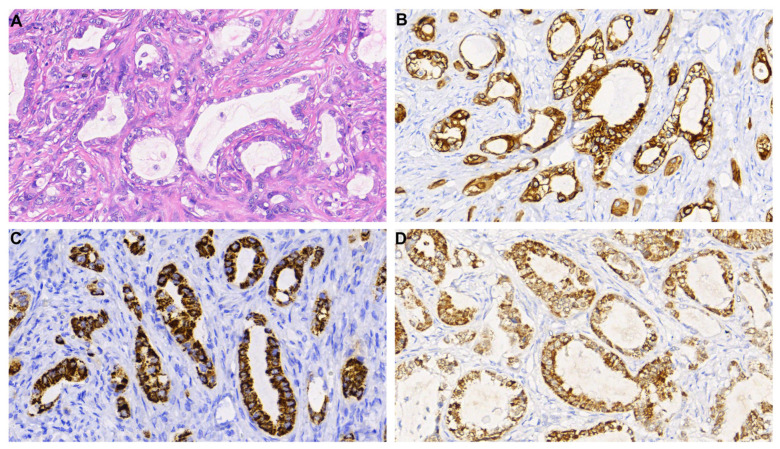
Histopathologic analysis of the brain metastasis tumour sample. Hematoxylin and eosin-stained histological sections show adenocarcinoma, comprised cells with increased nuclear/cytoplasmic ratio and prominent nucleoli forming glands (**A**). Neoplastic cells display strong cytoplasmic positivity for cytokeratin 7 (**B**), strong cytoplasmic positivity for HEP-PAR (**C**), and both nuclear and cytoplasmic staining for TTF1 (**D**). Magnification: 300×.

The histopathological analysis was also performed and revealed an epithelial neoplasia composed of cells with rounded, vesicular nuclei and eosinophilic cytoplasm, forming glands. There were extensive areas of necrosis. Immunostains (Figure 3) showed positivity for cytokeratin 7 (CK7), with cytoplasmic and nuclear positivity for thyroid transcription factor 1 (TTF1), cytoplasmic positivity for both napsin A, and hepatocyte antigen (Hep-Par). Cytokeratin 20 was not expressed by tumour cells. The immunoprofile favoured a metastatic adenocarcinoma with hepatoid features.

**Figure 4:**
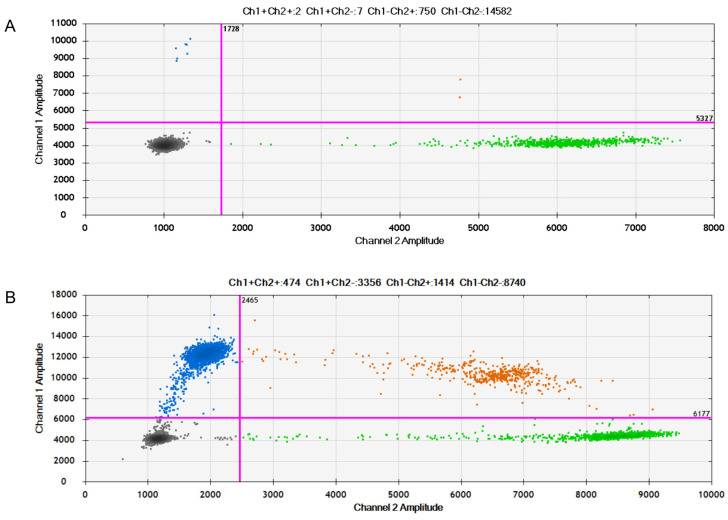
Two-dimensional scatter plot presenting four clusters obtained with mutant *K-RAS* and its wild-type allele. (**A**) Analysis of ctDNA extracted from patient’s plasma. (**B**) Analysis of ctDNA extracted from patient’s brain tumour mass. In both graphs (**A**,**B**), the fluorescence of channel 1 (FAM) is plotted against the fluorescence of channel 2 (HEX) for each drop (colour: blue, black, orange, or green). Drops are grouped into the following groups: FAM negative, negative HEX (doubly negative drops—black marking); FAM positive, HEX negative (positive drops for the sample with mutation—blue marking); FAM negative, HEX positive (positive drops for wild-type sample—green marking); FAM positive, HEX positive (doubly positive drops, containing both wild-type and mutated DNA—orange labelling). The ddPCR™ *KRAS G12/G13* Screening Kit (Bio-Rad’s catalogue number 1863506) detects seven *K-RAS* mutations: G12A, G12C, G12D, G12R, G12S, G12V, G13D (blue labelling), without discriminating each one of them.

We next investigated whether the ctDNA in the patient’s plasma could be detected and analysed using Droplet Digital PCR (ddPCR) following the methodology described in our previously published study [19]. Briefly, presurgical plasma samples were obtained and analysed for the presence of ctDNA via ddPCR using Bio-Rad’s mutation-specific assays (i.e., Bio-Rad’s ddPCR G12/13 screening kit able to detect seven KRAS mutations: G12A, G12C, G12D, G12R, G12S, G12V, G13D). The liquid biopsy revealed the presence of K-RAS mutations in the samples analysed, which could be any of the G12/13 mutations detected in the assay. The two-dimensional scatter plot shows blue and orange dots representing, respectively, mutant-only and wild-type plus mutant K-RAS ctDNA in the patient’s plasma (Figure 4A), with a mutation frequency of 1.2%. In addition, we performed the same analysis on the patient’s tumour sample that was removed during surgery followed by formalin fixation and paraffin embedding. Interestingly, we found the same K-RAS mutations tested in the plasma sample, but at a much higher rate, 68.8% (Figure 4B).

## 3. Discussion

There is not enough knowledge regarding pathways that control blood–brain barrier (BBB) permeability in the normal brain versus brain tumours; however there is consensus that an important step during the metastatic cancer cell journey to the brain is the invasion through the BBB [20]. The most frequent primary tumours that metastasize to the brain are lung, breast, and melanoma, being magnetic resonance imaging (MRI) and CT scan, commonly used in the initial diagnosis of brain tumours [21]. Regarding the molecular profile of brain metastasis, it was previously observed that *K-RAS* mutations were significantly increased in primary lung tumours metastasized to the brain via next-generation sequencing (NGS) analysis [22]. Interestingly, the ddPCR performance has demonstrated more accuracy when compared to NGS in distinct tumour analysis [23]. The main difference between NGS versus ddPCR techniques is that the latter is mainly restricted to the detection of known mutations, with limited capability of detecting several mutations per assay, whereas NGS more broadly detects multiple specific genetic changes at once but cannot efficiently detect mutation frequencies below 1% as the ddPCR does [24].

There are insufficient studies evaluating the precision of the ddPCR analysis performed in plasma versus brain tumour tissues, probably due to the difficulty in obtaining enough levels of nucleic acids extracted from plasma for analysis. In addition, only a small portion of cfDNA contains ct-DNA. Thus, the present report contributes to the field since it indicates the high sensitivity of the ddPCR technology in identifying low-frequency mutations both in the plasma and in the tumour tissue of the same patient. Our analysis showed the presence of *K-RAS* mutations in both samples, which are not frequently found in brain tumours but have been reported in lung hepatoid adenocarcinoma [25], a tumour that was also found in the patient analysed. In addition, lung cancers frequently harbour *K-RAS* mutations, and it is also one of the most common origins of brain metastases [22], which could potentially explain the present finding in the tumour tissue analysed. Besides, it has been shown that *K-RAS* alterations found in plasma are significantly predictive of *K-RAS* tumour status with significant tumour and plasma status consensus [26,27].

Although one limitation of ctDNA detection relies on the low abundance of ctDNA fragments in plasma samples making its detection difficult, the ddPCR method can successfully detect low-frequency mutations, even below 1% [28]. In our study, we were able to detect a *K-RAS* mutation frequency of 1.2% compared to 68.8% in the tumour sample analysed. Thus, the ddPCR method could be potentially employed to detect metastatic events and serve as a monitoring tool for the evaluation of gene mutations, such as *K-RAS*, in both primary and metastatic brain tumours. The major limitation of our report is that we were unable to obtain plasma samples after the brain tumour’s surgical resection, which could have been useful to check if the levels of circulating *K-RAS* ctDNA were affected after surgery.

Even though liquid biopsies have been established in various clinical settings, there are still important validations to be made in the brain tumours’ scenario, including noninvasive diagnosis of different brain tumour types, prognosis, disease monitoring and prediction of minimal residual disease.

## 4. Conclusions

Although the evaluation of ctDNA alone cannot yet bypass the need for tissue biopsies, it may be complementary to other diagnostic and disease monitoring tools, such as in brain metastases. A study including large numbers of paired samples will be important to further confirm this finding and to support liquid biopsy as a noninvasive and reliable screening method that could potentially benefit patients with brain tumours.

## Data Availability

Not applicable.
